# Supplementary material for: Direct X-ray photoconversion in flexible organic thin film devices operated below 1 V
Source: Nat Commun. 2016 Oct 6;7:13063. doi: 10.1038/ncomms13063 (PMC5059709; doi:10.1038/ncomms13063)
Supplement: Supplementary Information — Supplementary Figures 1-7, Supplementary Notes 1-4 and Supplementary References [file ncomms13063-s1.pdf]

Supplementary Figures

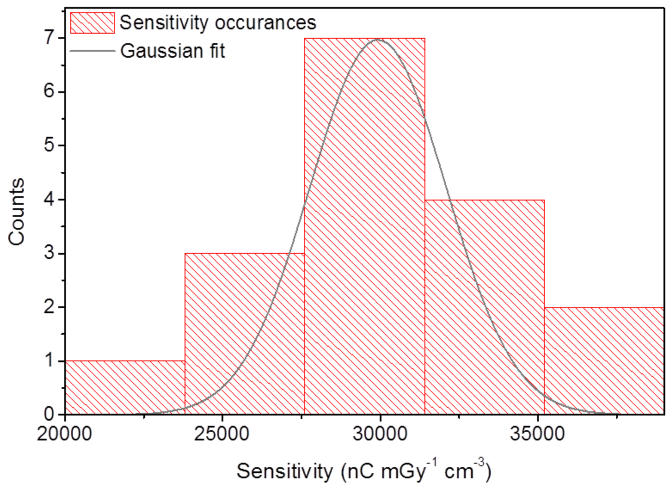

Supplementary Fig.1: Histogram of sensitivity values measured over 17 devices. Data refers to experiments performed in a dose rate range of (10÷20) mGy s^{-1}.

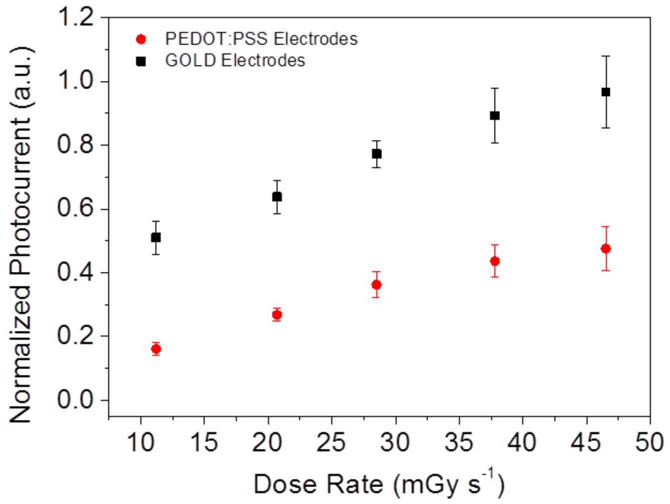

Supplementary Fig.2: Plots of the X-ray photocurrent in function of dose rate of TIPS-pentacene thin film device with gold electrodes (black squares) and of a full-organic one with PEDOT:PSS electrodes (red circles). Both the devices are biased with 1 V and have the same electrode geometry.

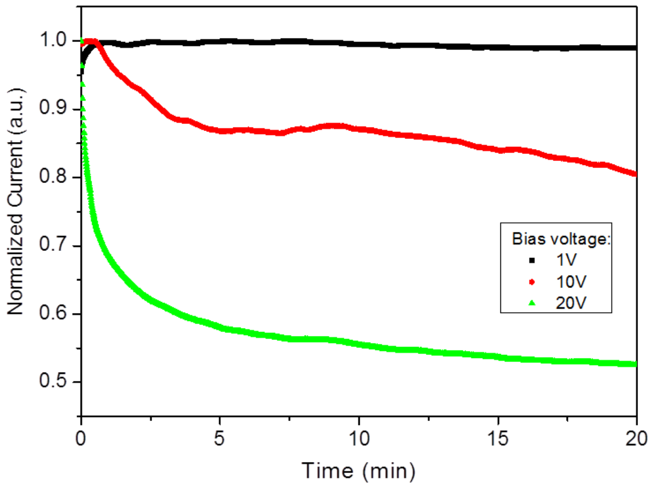

Supplementary Fig. 3: Normalized current decreasing with a fixed applied voltage of 1V (black squares), of 10V (red circles) and of 20V (green triangles) in function of time. The plot clearly shows that the current is almost stable within 20min of continuous biasing at 1V, while at higher bias voltages the current drops quickly, decreasing of about 50% at 20V.

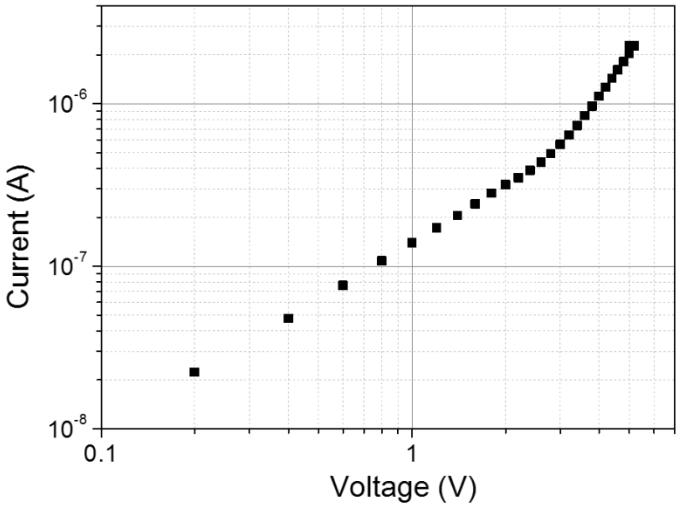

Supplementary Fig.4: Current vs. Voltage characteristics of the device in dark. At voltages higher than 2 V the plot becomes non-ohmic due to the inception of space charge effect[4];

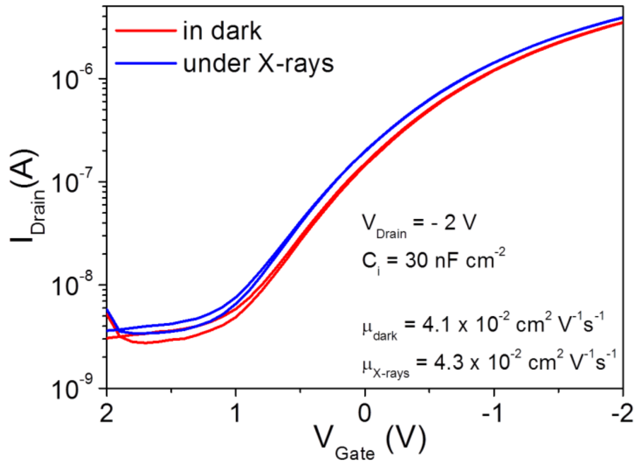

Supplementary Fig. 5: Transfer characteristics in dark and under X-ray irradiation (Mo-tube at 35 keV) of a TIPS-pentacene based transistor in saturation regime (V_{drain}=-2 V). In the inset the hole mobility values, calculated from the two curves, are reported.

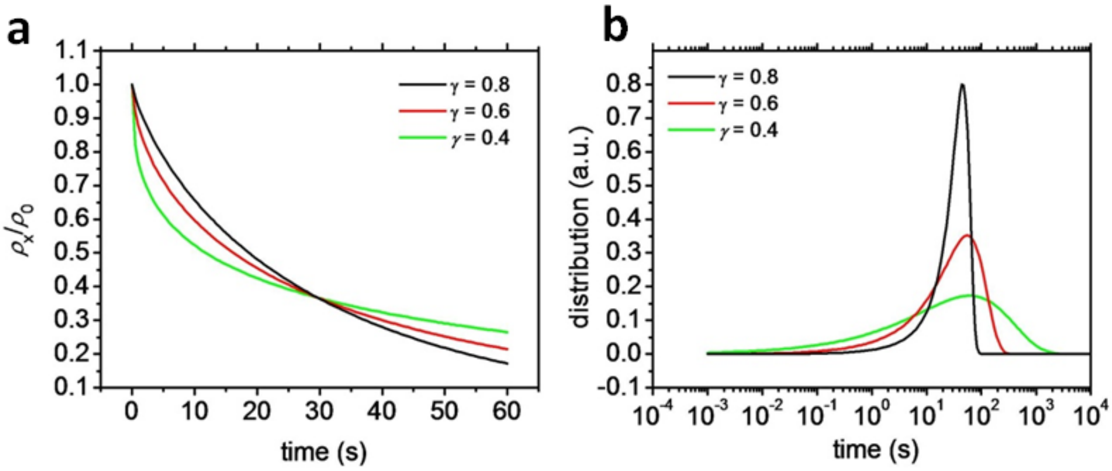

Supplementary Fig. 6: (a) Stretched exponentials and (b) their inverse Laplace Transform describing the distribution of time-scales involved in X-ray photocurrent dark relaxation.

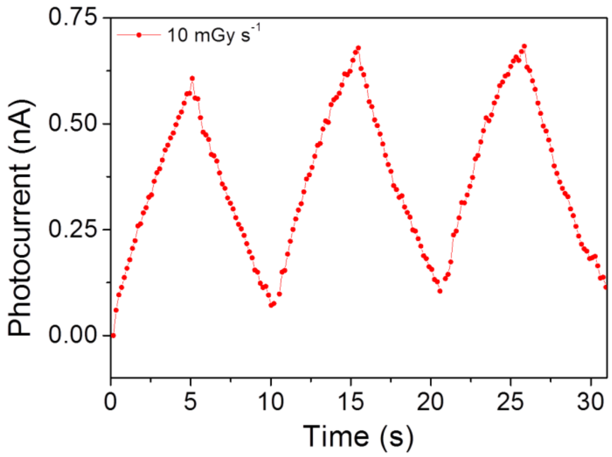

Supplementary Fig. 7: X-ray induced photocurrent signal recorded at a bias voltage of 0.2 V, upon three on/off switching cycles of 5 s each, of a monochromatic synchrotron X-ray beam at 17 keV. For an exposure time of 5 s, the signal does not reach saturation.

Supplementary Notes

Supplementary Note 1: Calculation of photon flux

The incident photon flux is calculated as $\Phi_0 = \frac{D}{c_\mathrm{m}}\frac{A}{E_\mathrm{ph}}$, with D indicating the X-ray dose rate, c_m the air attenuation coefficient, $A = 0.015\ \mathrm{cm}^2$ is the active area of the device and E_ph the photon energy.

The photon absorption rate Φ can be calculated from the Lambert-Beer equation:

$$\Phi = \Phi_0[1 - exp(-h/\lambda)]$$

where $\lambda = (c_\mathrm{mTIPS}\varrho)^{-1}$ is the attenuation length, with c_mTIPS indicating the mass attenuation coefficient of TIPS-pentacene, ϱ = 1.1 g cm^{-3} the TIPS-pentacene density, h=100 nm. In the calculation we consider the limiting case $\lambda \gg h$. A radiation dose rate of 19.3 mGy s^{-1} at 17 keV (the same experimental conditions of data reported in Fig. 1d), corresponds to c_m= 1.14 cm^2 g^{-1} and c_mTIPS= 1.34 cm^2 g^{-1} (calculated with the XCOM code[1]).

Supplementary Note 2: Interpretation of fit parameters α, γ, ρ_0

The stretched exponential behavior of the X-ray photocurrent transient, described by parameters α, γ, ρ_0, is characteristic for transport in dispersive systems[2]. Instead of a single level determining carrier recombination as described by an exponential kinetics, a distribution of states with different recombination times is involved. Consequently, α denotes an effective time-scale on which dark relaxation (after X-ray irradiation turned off) happens. Meaning that, when starting with an initial charge density $\rho_\mathrm{X}(t=0)=\rho_0$, then $\rho_\mathrm{X}(t=\alpha^{1/\gamma})= \rho_0/e$ (see Supplementary Fig. 6a). The parameter γ describes the width of the distribution of relaxation time-scales α_i. For $\gamma=1$, only one time-scale is present and the dark relaxation kinetics follows an exponential behavior. Instead, for $\gamma < 1$ the distribution gets broader. For the sample analyzed we observe $\gamma=0.61$. For a broad distribution, a stretched exponential behavior results as initially a fast relaxation occurs due to holes recombining with electrons escaping from shallower traps. As the charge density reduces, the recombination process slows down, as holes have to recombine with electrons escaping from deeper traps. The distribution of time-scales can be calculated by the inverse Laplace transform of the stretched exponential relaxation kinetics[2,3].

The distribution observed here, combined with some other characteristic distributions at different γ values for comparison, are shown in Supplementary Fig. 6.

Supplementary Note 3: Estimation of the photoconductive gain of the detector

Employing equation (2) we can calculate the carrier concentration ρ_X in the steady-state considering the photocurrent experimentally recorded after 60 s of exposure to a 19.3 mGy s^{-1} dose rate of 17 keV X-ray radiation (the experimental condition of the measurement reported in Fig.1d), $\Delta I_\mathrm{PG}\approx 3$ nA:

$$\Delta I_\mathrm{PG}=Wh\rho_\mathrm{X}(t)\mu E \Rightarrow \rho_X=\frac{\Delta I_\mathrm{PG}L}{WhV\mu}\approx 2.35\times 10^{-5}\mathrm{C\ cm^{-3}}$$

The parameters used for the calculation are: channel width and length respectively of W = 48 mm and L = 30 μm and radiation dose rate, bias voltage V=0.2 V and $\mu=0.04\ \mathrm{cm}^2$ V^{-1} s^{-1}.

The carrier lifetime results:

$$\tau_{\mathrm{r}} = \frac{\alpha}{\gamma}\left[\alpha \ln\left(\frac{\rho_0}{\rho_{\mathrm{X}}}\right)\right]^{\frac{1-\gamma}{\gamma}} = 29.4 \text{ s}$$

Obtained considering the fitting parameters $\alpha = 7.9$ s, $\gamma = 0.61$ and $\rho_0 = 3.7 \times 10^{-5} \mathrm{C\ cm^{-3}}$.

The transit time results:

$$\tau_{\mathrm{t}} = \frac{L^2}{V\mu} = 1.1 \text{ ms}$$

The photoconductive gain thus results:

$$G = \frac{\tau_{\mathrm{r}}}{\tau_{\mathrm{t}}} = \frac{29.4}{1.1 \times 10^{-3}} = 2.6 \times 10^4$$

Supplementary Note 4: Sensitivity of organic X-ray detectors in dependence of exposure time to the radiation and implicit relation for dose-rate dependence

Following equation (3) in the limiting case of short time exposure to the radiation after Taylor expansion:

$$\rho_{\mathrm{X}}(t) \approx \left.\frac{\partial \rho_{\mathrm{X}}(t)}{\partial t}\right|_{\rho_{\mathrm{X}}=0} t = \frac{\Phi n q}{A h} t$$

Substituting in equation (2):

$$\Delta I_{\mathrm{PG}} = W h \rho_{\mathrm{X}}(t) \mu E = \frac{W h \mu V}{L} \frac{\Phi n q}{A h} t = \frac{n q t}{\tau_{\mathrm{t}}} \Phi = \frac{t}{\tau_{\mathrm{t}}} \frac{n q A}{c_{\mathrm{m}}} \left(1 - e^{-h/\lambda}\right) D = \frac{t}{\tau_{\mathrm{t}}} S_{\mathrm{CC}} D$$

Using: $\Phi = \frac{DA}{c_{\mathrm{m}} E_{\mathrm{ph}}}\left(1 - e^{-h/\lambda}\right)$; $LW = A$; $\tau_{\mathrm{t}} = \frac{L^2}{\mu V}$; $I_{\mathrm{CC}} = S_{\mathrm{CC}} D = n q \Phi$;

with: $S_{\mathrm{CC}} = \frac{n q A}{c_{\mathrm{m}} E_{\mathrm{ph}}}\left(1 - e^{-h/\lambda}\right)$

On the other hand, in the steady-state ($\partial \rho_{\mathrm{X}} / \partial t = 0$):

$$0 = \frac{\Phi nq}{Ah} - \frac{\rho_X(t)}{\tau_r(\rho_X)} = \frac{Wh\mu V}{L}\frac{\Phi nq}{Ah} - \frac{Wh\mu V}{L}\frac{\rho_X(t)}{\tau_r(\rho_X)} = \frac{D}{c_m E_{ph}}\left(1 - e^{-\frac{h}{\lambda}}\right)\frac{nq}{h} - \frac{\Delta I_{PG}}{\tau_r(\rho_X)}$$

$$= \frac{Dnq}{c_m E_{ph}}\left(1 - e^{-\frac{h}{\lambda}}\right)\frac{LW}{\tau_t} - \frac{\Delta I_{PG}}{\tau_r(\rho_X)}$$

$$\Delta I_{PG} = \frac{nqLW}{c_m E_{ph}}\left(1 - e^{-\frac{h}{\lambda}}\right)\frac{\tau_r(\rho_X)}{\tau_t}D = S_{CC}\frac{\tau_r(\rho_X)}{\tau_t}D \qquad \text{(Supplementary Equation 1)}$$

Combining equations (2) and (4) we obtain:

$$\tau_r(\rho_X) = \tau_r(\Delta I_{PG}) = \frac{\alpha}{\gamma}\left[\alpha \ln\left(\frac{I_0}{\Delta I_{PG}}\right)\right]^{\frac{1-\gamma}{\gamma}}$$

Inserting this into Supplementary Equation 1:

$$D = c\Delta I_{PG}\left[\ln\left(\frac{I_0}{\Delta I_{PG}}\right)\right]^{\frac{\gamma-1}{\gamma}} \qquad \text{(Supplementary Equation 2)}$$

with $c = \frac{\tau_t \gamma}{S_{CC}\alpha^{1/\gamma}}$

Supplementary Equation 2 allows to plot D as a function of ΔI_{PG}. Parameters c, I_0 and γ are determined by a least square fit of the experimental data.

Supplementary References

1. M.J. Berger et al., XCOM: photon cross section database, http://www.nist.gov/pml/data/xcom/index.cfm (1998).
2. D. Redfield and R. H. Bube. *Photoinduced Defects in Semiconductors*. (Cambridge University Press, 1996).
3. Luo, J. *et al.* Transient photoresponse in amorphous In-Ga-Zn-O thin films under stretched exponential analysis. *J. Appl. Phys.* **113,** 153709 (2013).
4. Lampert, M. A. & Mark, P. *Current injection in solids*. (Academic Press, 1970).
